# 

**Published:** 1891-10

**Authors:** 


### TABLE OF CONTENTS.

ORIGINAL COMMUNICATIONS. PAGE

What Value has Argenti Nitras as a Therapeutic Agent in Dentistry? By Dr. E. A. Stebbins, Shelburne Falls, Mass. . . . . . . . . . . . . . 661

Pathological Conditions Produced by Galvanic Action between Dissimilar Metals in the Treatment of Caries of the Teeth. By George W. Whitefield, M.D., D.D.S., Evanston, Ills. . . . . . . . . . . . . . 671

Vitality as a Germicide. By Thomas Fillebrown, M.D., D.M.D., Boston . . . . . . . . . . . . . . . . . . . . . . . . . . . . 678

The Rheumatic and Gouty Diathesis as Manifested in Diseases of the Peridental Membrane. By John S. Marshall, M.D. . . . . . . . . . 690

REPORTS OF SOCIETY MEETINGS.

New York Odontological Society . . . . . . . . . . . . . . . . . . . . 694

American Medical Association . . . . . . . . . . . . . . . . . . . . 707

American Dental Association . . . . . . . . . . . . . . . . . . . . 717

American Academy of Dental Science . . . . . . . . . . . . . . . . . . 728

EDITORIAL.

Criticism of Dental Colleges . . . . . . . . . . . . . . . . . . . . . 731

Nitrate of Silver.—Stebbins . . . . . . . . . . . . . . . . . . . . . 734

To the "Dental Review" . . . . . . . . . . . . . . . . . . . . . . . 734

Deposit Plates . . . . . . . . . . . . . . . . . . . . . . . . . . 735

Bibliography . . . . . . . . . . . . . . . . . . . . . . . . . . . 735

DOMESTIC CORRESPONDENCE.

A Communication from the Dental Protective Association of the United States . . . . . . . . . . . . . . . . . . . . . . . . . . . . . 737

CURRENT NEWS.

The Ohio State Dental Society . . . . . . . . . . . . . . . . . . . . 739

The Fifth, Sixth, Seventh, and Eighth District Dental Societies of New York State . . . . . . . . . . . . . . . . . . . . . . . . . . . 739

Post-Graduate Association of the United States . . . . . . . . . . . . 740

New Hampshire Dental Society . . . . . . . . . . . . . . . . . . . . 740

